# Early impairment of coronary microvascular perfusion capacity in rats on a high fat diet

**DOI:** 10.1186/s12933-015-0312-2

**Published:** 2015-11-17

**Authors:** Judith van Haare, M. Eline Kooi, Hans Vink, Mark J. Post, Jurgen W. G. E. van Teeffelen, Jos Slenter, Chantal Munts, Hanneke Cobelens, Gustav J. Strijkers, Dennis Koehn, Marc van Bilsen

**Affiliations:** Department of Physiology, CARIM School for Cardiovascular Diseases, Maastricht University, P.O. Box 616, 6200 MD Maastricht, The Netherlands; Department of Radiology, CARIM School for Cardiovascular Diseases, Maastricht University, P.O. Box 616, 6200 MD Maastricht, The Netherlands; Department of Cardiology, CARIM School for Cardiovascular Diseases, Maastricht University, P.O. Box 616, 6200 MD Maastricht, The Netherlands; Biomedical Engineering and Physics, Academic Medical Center, P.O. Box 22700, 1100 DE Amsterdam, The Netherlands; Pie Medical Imaging, P.O. Box 1132, 6201 BC Maastricht, The Netherlands

**Keywords:** Obesity, Endothelial dysfunction, Myocardial perfusion, Cardiac function, Glycocalyx

## Abstract

**Background:**

It remains to be established if, and to what extent, the coronary microcirculation becomes compromised during the development of obesity and insulin resistance. Recent studies suggest that changes in endothelial glycocalyx properties contribute to microvascular dysfunction under (pre-)diabetic conditions. Accordingly, early effects of diet-induced obesity on myocardial perfusion and function were studied in rats under baseline and hyperaemic conditions.

**Methods:**

Rats were fed a high fat diet (HFD) for 6 weeks and myocardial microvascular perfusion was determined using first-pass perfusion MRI before and after adenosine infusion. The effect of HFD on microcirculatory properties was also assessed by sidestream darkfield (SDF) imaging of the gastrocnemius muscle.

**Results:**

HFD-fed rats developed central obesity and insulin sensitivity was reduced as evidenced by the marked reduction in insulin-induced phosphorylation of Akt in both cardiac and gastrocnemius muscle. Early diet-induced obesity did not lead to hypertension or cardiac hypertrophic remodeling. In chow-fed, control rats a robust increase in cardiac microvascular perfusion was observed upon adenosine infusion (+40 %; p < 0.05). In contrast, the adenosine response was abrogated in rats on a HFD (+8 %; N.S.). HFD neither resulted in rarefaction or loss of glycocalyx integrity in skeletal muscle, nor reduced staining intensity of the glycocalyx of cardiac capillaries.

**Conclusions:**

Alterations in coronary microcirculatory function as assessed by first-pass perfusion MRI represent one of the earliest obesity-related cardiac adaptations that can be assessed non-invasively. In this early stage of insulin resistance, disturbances in glycocalyx barrier properties appeared not to contribute to the observed changes in coronary microvascular function.

## Background

Obesity is one of today’s major worldwide health problems and is a primary risk factor for type 2 diabetes and cardiovascular disease [1, 2]. In particular, abdominal obesity is associated with insulin resistance [3, 4]. Several studies reported altered microvascular function and microvascular remodeling in the skin and skeletal muscle of obese subjects [5–7]. The changes in microvascular properties observed under these conditions include impaired vasomotor activity, increased permeability, reduced capillary recruitment and rarefaction [8–12]. More recent studies also point to abnormalities in glycocalyx function contributing to the microvascular dysfunction [13–15]. The endothelial glycocalyx contributes to the vasculoprotective effects of the vessel wall by mediating nitric oxide (NO) release, by acting as a permeability barrier, and by inhibiting platelet and leukocyte adhesion [16–18]. Indeed, a reduction of microvascular glycocalyx dimensions has been observed in patients with increased cardiovascular risk [15]. In mice with diet-induced obesity, microcirculatory glycocalyx dysfunction in skeletal muscle was observed [13]. To date information on whether the abnormalities in microvascular function in obese and insulin resistant subjects also involve the coronary microcirculation is scarce and often conflicting [19–25]. As far as the glycocalyx of the coronary circulation is concerned, it has been shown that the glycocalyx is shedded in case of highly injurious conditions like myocardial ischemia/reperfusion [26]. If glycocalyx integrity also gets perturbed under less injurious conditions, as evoked by diet-induced obesity, is entirely unknown.

Accordingly, in the present study it was explored if diet-induced obesity affects cardiac function and coronary microvascular perfusion at an early stage, and if so, if this was linked to compromised glycocalyx function. Obesity was induced by challenging rats with a high fat diet (HFD; 60 % kcal fat) for 6 weeks [14, 27, 28] and insulin resistance was monitored by measuring myocardial Akt phosphorylation. Myocardial perfusion was determined under baseline and hyperaemic conditions as induced by adenosine, using first-pass perfusion MRI, a non-invasive method that is increasingly applied in the clinic for evaluation of the myocardial perfusion status [29, 30]. Cardiac function was assessed using cine MRI. To evaluate the effects of HFD on glycocalyx properties more directly, the microcirculation of the gastrocnemius was visualised with a Sidestream Dark-Field (SDF) camera in the same animals. HFD-induced changes in cardiac phenotype were assessed by qPCR and (immuno)histological analysis.

Collectively, the present findings show that the adenosine-evoked increase in myocardial microvascular perfusion is blunted in diet-induced obese rats, mainly due to an already increased basal perfusion. We found no evidence that, in this early stage of insulin resistance, changes in glycocalyx properties contributed significantly to the observed changes in coronary microvascular function.

## Methods

### Animals and diet

All animal experiments were performed according to a protocol approved by the Animal Ethics Care and Use committee of Maastricht University (AEC protocol number: 2010-122). 10 weeks old male Wistar Hannover rats (n = 18; body weight 300–324 g; Harlan Laboratories, Horst, The Netherlands) were housed in standard cages with 2 rats per cage, in a temperature-controlled room (~23 °C; 40–60 % humidity) under a 12/12 h light/dark cycle at the animal facility of Maastricht University. Rats received standard chow (Ssniff GmbH, Soest, Germany, containing on caloric basis 9 % fat, 58 % carbohydrates, and 33 % protein) for 6 weeks (n = 9) or HFD (D12492; Research Diets, New Brunswick, containing on caloric basis 60 % fat, 20 % carbohydrate, and 20 % protein), also for 6 weeks (n = 9). All animals received diet and water ad libitum.

Every week, body weight was determined and blood pressure was measured by the tail cuff method using a CODA non-invasive blood pressure monitoring system (Kent Scientific, Torrington, CT). Blood was sampled from the saphenous vein in the non-fasted, conscious animals to determine glucose and plasma insulin levels. Blood glucose (~5 µl) was measured with a glucose meter (Ascencia Contour, Bayer, Mijdrecht, The Netherlands). Blood was collected using 75 µl glass capillary tubes (Hirschmann, Eberstadt, Germany). The capillary tubes were centrifuged, plasma hematocrit levels were determined (MSE, micro-haematocrit reader), and plasma was collected to measure plasma insulin levels with an Ultrasensitive Insulin ELISA kit according to the manufacturer’s protocol (ALPCO Diagnostics, Salem, NH).

A separate set of control rats (n = 9) and HFD rats (n = 9) was used to perform an intravenous insulin tolerance test (AEC protocol number: 2011-139). The rats were housed under the same conditions as described before.

### MR imaging

At the day of experiment, non-fasted rats were subcutaneously injected with 0.05 mg/kg of buprenorphine about 30 min before surgery, and were anesthetized with isoflurane (induction chamber: 4.5 % isoflurane and 1 % oxygen, during experiment: 2 % isoflurane and 0.4 % oxygen). The animal was put in a prone position and body temperature was maintained at ~37 °C by the use of a heating pad. During anesthesia, a cannula was inserted in the jugular vein for infusion of adenosine for stress measurements. For the infusion of the contrast agent for MRI, both left and right femoral vein were cannulated. After surgery, the anesthetized rats were positioned prone and horizontally in an animal holder on a heating pad in the magnet. Body temperature was monitored by a rectal probe. ECG electrodes were positioned on the right front leg and left hind leg and connected to an MR compatible small animal monitoring system (SA Instruments, Stony Brook, NY, USA). Respiratory rate was continuously monitored. Magnetic resonance imaging to determine myocardial function and perfusion was performed during baseline conditions and during intravenous adenosine infusion (3 mg/ml; 1.5 ml/h).

#### Cine MRI

Magnetic resonance imaging was performed on a 7.0 Tesla Bruker Biospec 70/30 USR animal scanner (Bruker Biospin, Ettlingen, Germany) equipped with a quadrature volume coil. Sequential multislice short-axis cine MRI was performed to assess systolic function of the left ventricle. First, a bright blood cine MR image with 10 cardiac phases was recorded in 4-chamber view (4CH) using a retrospectively self-gated protocol (IntraGate, Bruker Biospin) as described previously [31]. The following acquisition parameters were used: pulse repetition time (TR) = 8 ms, echo time (TE) = 2.95 ms and flip angle = 10°. The acquired field of view was 50 × 50 mm^2^ with an acquired 256 × 256 matrix size with an imaging time of ~5 min. Perpendicular to the 4CH view, a long axis view (LA) was acquired using the same self-gated protocol. This imaging slice was oriented as a plane through the mitral valve and the apex. Perpendicular to this LA and 4CH view, two short axis slices (SA) were positioned just below the mitral valve and just above the start of the papillary muscles. For both slices, the same self-gated protocol was used.

#### Myocardial first-pass perfusion MR imaging

A time-series of 300 short-axis images was acquired using a first-pass perfusion sequence, so that the first pass of the contrast agent through the heart was captured. A bolus of 150 µl Gadobutrol (200 mM; Gadovist, Bayer HealthCare, Berlin, Germany) was injected during the 15th time phase in the femoral vein, while scanning continued. For the mid-ventricular, short-axis first-pass perfusion images, an ECG-triggered, segmented saturation-recovery FISP sequence was used as described in detail elsewhere [32, 33]. A temporal resolution was reached of one image per four heartbeats. The prospective trigger delay δ_SAT_ with respect to the R-peak of the ECG-signal was adjusted to the heart rate and chosen such that the effective saturation time *T*_SAT_ was between 20 and 30 ms. The segment acquisition time *T*_ACQ_ was 25.76 ms. Additionally, the following acquisition parameters were used: TR = 1.61 ms, TE = 0.8 ms, field of view = 6 × 6 cm^2^, acquisition matrix = 64 × 64 (4 segments, acquired over four consecutive heart beats), slice thickness = 3 mm, 0.3 ms Gauss excitation pulse (α = 15°; BW = 9133 Hz) and a 2 ms Gauss saturation pulse (α = 90°; BW = 1370 Hz). Reconstructed pixel size was 469 × 469 µm^2^ after zero-filling to 128 × 128. Centric *k*-space filling was performed to ensure that low spatial frequency information was least influenced by cardiac motion.

### Imaging of skeletal muscle microcirculation

To determine the effect of HFD on glycocalyx barrier properties, the microcirculation of the gastrocnemius was visualised with a Sidestream Dark-Field (SDF) camera after the first-pass perfusion MR imaging. Three measurements were performed in each rat to visualise the microvessels on different positions in the gastrocnemius. The SDF camera is equipped with a 5x magnifying objective lens system-containing probe, imaging the red blood cells (RBCs) in the tissue-embedded microcirculation using green pulsed LED ring illumination [14, 34]. A micromanipulator was used for holding the SDF camera at the same position. Dimensions of the endothelial glycocalyx were estimated by imaging the microcirculation in the gastrocnemius using the SDF camera with integrated software (GlycoCheck BV, Maastricht, The Netherlands) for automatic analysis of the video recordings as previously described [35, 36].

After imaging of the muscle microcirculation, when the animal is still under anesthesia, the rat was sacrificed by cutting the diaphragm, and gastrocnemius were removed and immediately cut in two parts for fixation in a 4 % formaldehyde solution and in Tissue-Tek (O.C.T. Compound) for cryostat sectioning. The heart was also fixated for histological analysis. For only four control rats and four rats on HFD, heart weight was determined, even as epidydimal and perirenal fat.

### Insulin tolerance test

After an overnight fast (10–12 h) an intravenous insulin tolerance test (IVITT) was performed in a separate set of chow-fed (n = 9) and HFD-fed rats (n = 9), to measure insulin-mediated glucose disposal. After analgesia and anesthesia (isoflurane 2 %) the femoral vein was cannulated. Baseline blood glucose concentration was measured via tail bleeding and 2 glass capillaries of blood were taken for baseline plasma insulin measurements. Next, rats received a bolus of glucose (1 g/kg, 0.5 g/ml, i.v.) to avoid the development of hypoglycemia after administering the bolus of insulin 30 min later (0.5 U/kg; 0.5 U/ml). Blood glucose and plasma insulin were measured as described above at the indicated time points. Rats were sacrificed before glucose infusion, and 5 min and 30 min after insulin infusion. Heart, gastrocnemius and soleus muscle were isolated, instantly frozen in liquid nitrogen, and stored at −80 °C.

### Western blotting

Tissues were homogenized, sonicated, and centrifuged in ice-cold SET buffer in the presence of phosphatase inhibitor (PhosSTOP, Roche) and protease inhibitor (Complete, Roche). Protein concentrations of the supernatants were determined using a BCA Protein Assay (Micro BCA Protein Assay Kit, Pierce). After electrophoresis samples were transferred to a PVDF membrane (Bio-Rad) and, after washing, incubated with a phospho-Akt(Ser473) rabbit antibody (Cell Signalling Technology, Beverly MA) and subsequently with anti-rabbit secondary antibody (Cell Signalling Technology) and with HRP substrate (SuperSignal West Femto Chemiluminescent Substrate, Pierce). After stripping (Restore Western Blot Stripping Buffer, Pierce) membranes were incubated with an Akt antibody (Cell Signalling Technology). Chemiluminescent signals were detected and quantified using the ChemiDoc XRS system (Bio-Rad). The ratios of pAkt/Akt were used to assess differences in insulin signalling.

### Data analysis

#### Cine MRI

All images were analysed in MRIcro. The end-diastolic volumes (EDV) and end-systolic volume (ESV) of the left ventricle were considered the largest and the smallest areas, respectively, of the LV cavity in each slice. For the analysis of cine MRI, the window width and level were manually adjusted to recognize the internal ventricular morphologic characteristics [31]. For the measurements of LV volumes, the whole cavity was manually selected in MRIcro. The papillary muscle was excluded from the LV volume during analysis. Length (L) of the LV on the LA view was defined as the longest distance from the apex to the valves. To calculate the left ventricular volumes (LVV), the modified simpson rule (SR) was used as described before [31]. Briefly, LVV = A_m_ × L/3 + (A_m_ + A_ρ_)/2 × L/3 + 1/3 × A_ρ_ × L/3. (A_m_, cross-sectional area of the LV cavity in the SA plane, ~1–2 mm below the mitral valve; A_ρ_, cross-sectional area of the LV cavity in the SA plane, approximately at the base of the papillary muscles).

Also the first derivative of LV volume over time (dV/dt) during the cardiac cycle was calculated for all rats. The change in volume and time during each of the cardiac cycle phase transitions of the 10 time frames was determined, during baseline and adenosine infusion [37].

Wall thickness of the left ventricle is calculated using the CAAS MRV FARM software package (version 2.1, Pie Medical Imaging, Maastricht, The Netherlands). In the short axis slice just below the mitral valve, the end-diastolic and end-systolic phase was marked. The endocardial and epicardial borders were manually delineated. Wall thickness is determined for the septum and the free wall, calculating the length of a segment between the epicardial and endocardial contours in the systolic and diastolic phase. For each of the segments, the distance between two contours is given in micrometers.

#### MR Perfusion analysis

In the first-pass perfusion data sets, manual segmentations were performed within the CAAS MRV FARM software package (version 2.1, Pie Medical Imaging, Maastricht, The Netherlands). Endocardial and epicardial borders were manually delineated. Contours were drawn in one frame of the slice and were copied automatically to the other, in total 300, frames. Contour edits were also copied to all frames in the slice and a visual check was performed to examine if the contours fit in all the images of the 300 time frames. In case the contours did not fit in an image, the contours were adjusted in that separate image. The LV cavity, or blood pool, was detected automatically if an endocardial contour was available. Papillary muscles were excluded from the LV cavity. To avoid partial volume effects within the myocardial and LV cavity regions of interests (ROI’s), a margin of 1 pixel was kept from the epicard and endocard contours.

After segmentation, signal intensity (SI)-time curves were obtained for the LV cavity and the myocardium. The relative upslope was calculated by dividing the maximum upslope of the myocardium by the maximum upslope of the LV cavity curve. The myocardium was divided in 6 segments, for each segment the relative upslope was calculated. For data analysis, the mean of these 6 segments was used. The upslope was calculated within a kernel of 2–3 time points for the LV cavity and 4–5 time points for the myocardium. The number of points within the kernel used for upslope calculations (e.g. 2–3 for the LV cavity and 4–5 for the myocardium) was adjusted for each signal intensity-time curve based on a visual inspection to acquire the best fit of the upslope.

### Imaging of skeletal muscle microcirculation

**Fig. 1:**
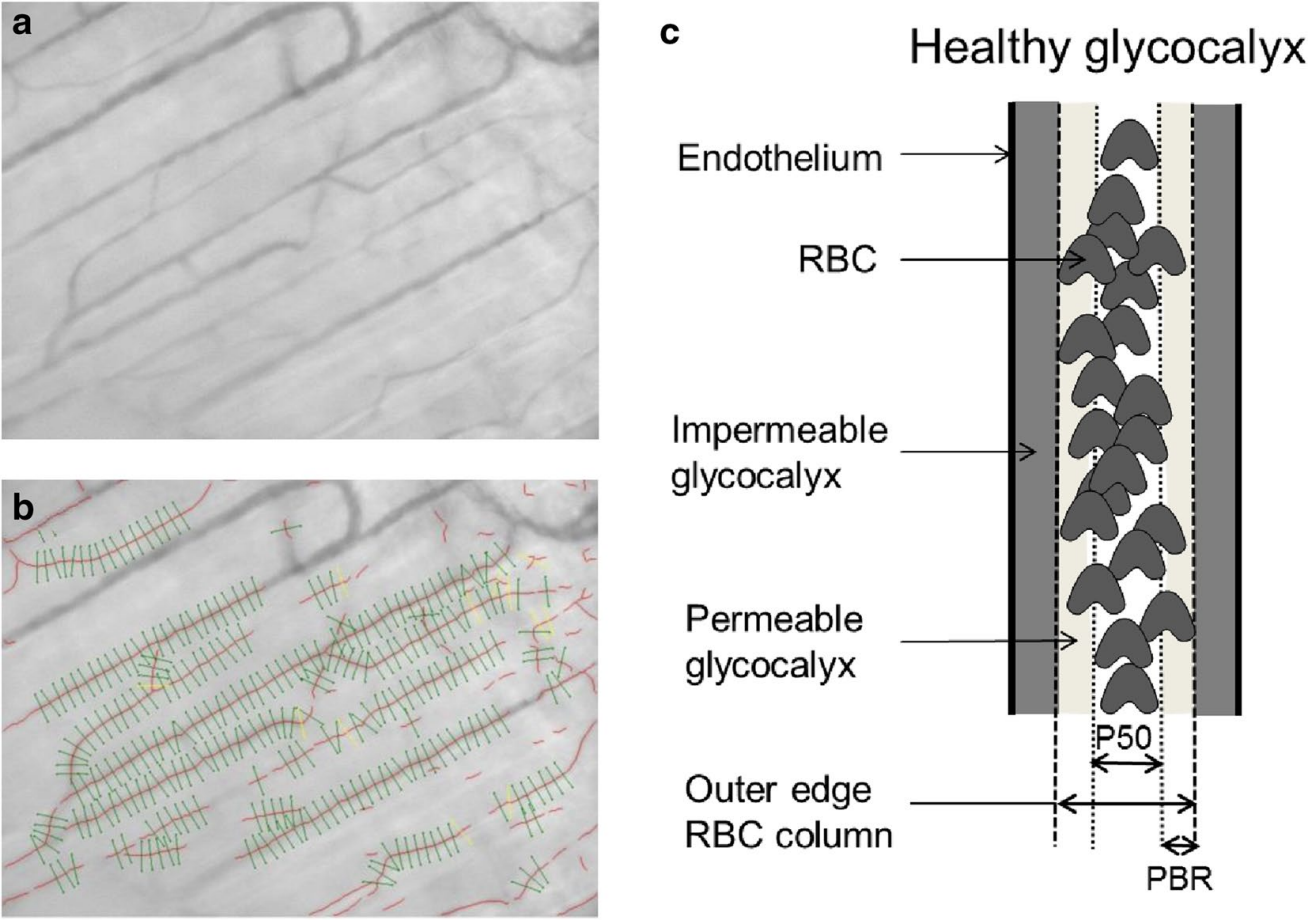
Imaging of skeletal muscle microcirculation. **a** Example of a single SDF image of rat gastrocnemius microcirculation. **b**, In each microvessel that fitted the criteria of analysis (a) in focus, b) had a sufficient length and were not close to bifurcation, and c) were continuously filled with RBCs and did not contain plasma gaps) lines were placed ± every 10 µm perpendicular to the vessel direction (as shown by lines in image). **c**, Schematic illustration of the perfused boundary region (PBR) in relation to the glycocalyx barrier properties in a blood vessel. The PBR covers the cell-permeable part of the glycocalyx, to which RBCs have limited access, while the cell-impermeable part cannot be accessed at all. The PBR is the main readout parameter of the gastrocnemius SDF imaging, and calculated from the median RBC column width (P50) and outer diameter of RBC perfused lumen

During video recording, the software automatically identifies all visible microvessels with a diameter between 5 and 25 µm and measurement sites perpendicular to the vessel direction were selected every 10 µm along the length of each microvessel (Fig. 1a, b). Data acquisition automatically started when image quality was within acceptable range, i.e. good red blood cell (RBC) and background contrast, in focus and without movement of the imaging unit. When data on a minimum number of 3000 measurement sites was obtained, data acquisition automatically stopped. Average duration of data acquisition was ~5 min. Movies consisted of 40 consecutive frames. In each frame, at each measurement site a total of 21 parallel (every ± 0.5 µm) intensity profiles were plotted. The RBC column width was automatically determined at each line for all 40 consecutive frames in a movie, revealing a total of 840 RBC column width measurements at a single measurement (21 profiles × 40 frames) [14]. RBC filling percentage was calculated by determining the number of measurement sites that were filled with a red blood cell divided by 840 (total number of measurement sites). Volume was calculated by RBC filling % multiplied by vessel density (number of perfused vessels per observed muscle area).

To calculate the perfused boundary region (PBR), which is defined as the distance between the median RBC column width and the estimated outer edge of the RBC column, the distribution of the RBC column width of each vascular segment is used (Fig. 1c). The cumulative distribution curve of the RBC column widths was constructed and used to determine median RBC column diameter (DP50). The maximum RBC column width is extrapolated from the 25th and 75th percentile values of the RBC column width distribution curve. The PBR is considered to reflect glycocalyx thickness based on the assumption that loss of integrity of the glycocalyx allows for deeper penetration of erythrocytes into the vessel wall, resulting in increased PBR values [38].

### Histological analysis

Paraffin-embedded hearts were sectioned in slices of 5 µm. After rehydration the sections were stained with 200 µl FITC-labeled lectin from triticum vulgaris (WGA-FITC; 50 µg/ml, Sigma) for 30 min in the dark at room temperature (20 °C), washed three times with PBS and mounted with antifade mounting medium (ProLong Gold; Thermo Fisher Scientific). Cardiac tissue slices were visualised and photographed using a Leica DFC350 FX digital camera (Leica, Rijswijk, The Netherlands) at 200× magnification with fixed exposure times and contrast settings (Leica DMI3000 microscope, Leica, Rijswijk, The Netherlands). Per animal, 5 pictures were analysed using Image J software. After defining a WGA-FITC signal intensity threshold for the control chow-fed animals, 50 capillaries per picture were selected and the average capillary WGA signal intensity was calculated for each animal.

For the CD68 staining, paraffin-embedded heart sections were washed in increasing concentrations of ethanol followed by PBS. Sections were incubated overnight with 150 µl CD68 antibody (1 mg/ml; AbD Serotec), followed by incubation with biotinylated Horse Anti-Mouse IgG Antibody, rat absorbed (HaM-RA; 0.5 mg/ml, Vector Labs) for 60 min. Sections were visualised using 0.5 % diaminobenzidine and 0.03 % hydrogen peroxidase, and counterstained with hematoxylin. The numbers of CD68-stained cells were determined in 10 randomly selected fields (0.25 × 0.25 mm; 400×).

Heart cryosections, sectioned in slices of 7 µm, were air-dried and fixated in 4 %-saline buffered formaldehyde solution, followed by incubation with Oil-Red-O (Fluka; Sigma-Aldrich) for 30 min. Sections were washed three times with MilliQ and counterstained with hematoxylin.

### Plasma lipid analysis

Cholesterol and triglyceride levels were measured in plasma samples of control and HFD rats. The enzymatic method to determine cholesterol level is based on the cleavage of cholesterol esters by cholesterol esterase to yield free cholesterol and fatty acids. Cholesterol oxidase then catalyzed the oxidation of cholesterol to cholest-4-en-3-one and hydrogen peroxidase. In the presence of peroxidase, the hydrogen peroxide formed affected the oxidative coupling of phenol and 4-aminophenazone to form a red quinone-imine dye. The color intensity of the dye formed is directly proportional to the cholesterol concentration and was determined by measuring the increase in absorbance (Cobas CHO2I and CHO2A, Roche Diagnostics, Mannheim, Germany).

The enzymatic triglyceride assay is based on using a lipoprotein lipase for the rapid and complete hydrolysis of triglycerides to glycerol followed by oxidation to dihydroxyacetone phosphate and hydrogen peroxide. The hydrogen peroxide produced then reacted with 4-aminophenazone and 4-chlorophenol under the catalytic action of peroxidase and a red dye was formed. The color intensity of the red dye formed is directly proportional to the triglyceride concentration and was measured photometrically (Cobas TRIGL, Roche Diagnostics, Mannheim, Germany).

### Gene expression analysis

Heart tissue fixated in Tissue-Tek (O.C.T. Compound) was sectioned in slices of 30 µm to collect tissue for RNA isolation. RLT buffer was added and heart tissue was homogenized. Total RNA was isolated from heart tissue using the RNeasy kit (Qiagen, Venlo, The Netherlands) according to the manufacturer’s protocol. RNA integrity and concentration were checked by means of the 260 nm/280 nm ratio, and cDNA synthesis was performed using 250 ng of RNA (Iscript cDNA synthesis kit, Biorad Inc., Hercules, CA, USA). Gene expression was analysed by quantitative PCR on an iCycler Real-Time PCR detection system using the iQ SYBR-green supermix (Biorad). The primer sequences are shown in Table 1. Results were normalized to the housekeeping gen cyclophilin-A, and relative changes in expression levels were calculated using the qBase analyser [37].

**Table 1:** Gene-specific primer sequences used for quantitative real-time PCR

Gene	Forward primer	Reverse primer
α-Skeletal actin (αSKA)	TGAGACCACCTACAACAGCA	CCAGAGCTGTGATCTCCTTC
Angiopoietin-like 4 (Angptl4)	CCTTTCCCTGCCCTTCTCTAC	AGGCTCTTGGCACAGTTAAGG
Atrial natriuretic factor (ANF)	AGCGAGCAGACCGATGAAGC	GCAGAGTGGGAGAGGTAAGGC
Cluster of differentiation 68 (CD68)	TAATACGACTCACTATAGGG	TAGAAGGCACAGTCGAGG
Cyclophilin-A (Cyclo)	CAAATGCTGGACCAAACACAA	TTCACCTTCCCAAAGACCACAT
Endothelial nitric oxide synthase (eNOS)	TGCTGCCCGAGATATCTTCAGT	GGCTGCCTTTTTCCAGTTGTTC
Intercellular adhesion molecule (ICAM)	AAACGGGAGATGAATGGTACCTAC	TGCACGTCCCTGGTGATACTC
Monocyte chemoattractant protein-1 (MCP1)	ATGCAGTTAATGCCCCACTC	TGCTGCTGGTGATTCTCTTG
Pyruvate dehydrogenase kinase 4 (PDK4)	GCATTTCTACTCGGATGCTCATG	CCAATGTGGCTTGGGTTTCC
Vascular adhesion molecule (VCAM)	GGCTCGTACACCATCCGC	CGGTTTTCGATTCACACTCGT

### Statistical analysis

All data were presented as mean ± standard error of the mean (SEM). Statistical differences between the control group on normal chow and the HFD group were tested using an unpaired or paired sample *t* test, a repeated measurements analysis of variance (ANOVA) or a one-way ANOVA. A P value of <0.05 was considered statistically significant.

## Results

### Animal characteristics

**Fig. 2:**
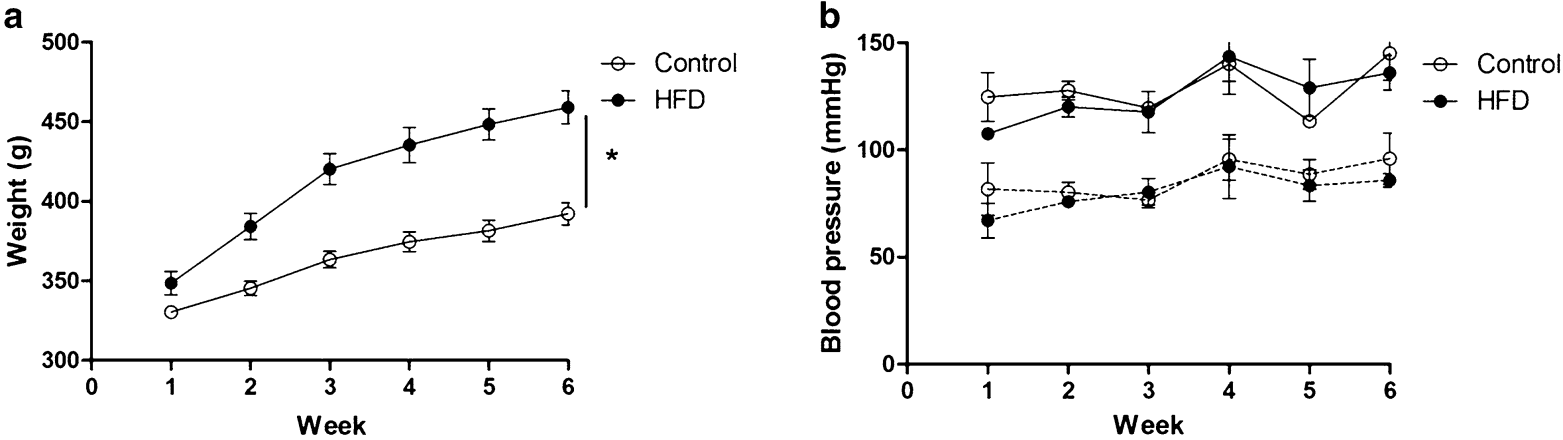
Body weight and systolic and diastolic blood pressure of the control and HFD group. **a** Body weight was significantly higher for rats feeding a HFD (n = 9, *filled circles*) for 6 weeks compared to control rats (n = 9, *open circles*). **b** Neither systolic (*solid line*) nor diastolic (*dashed line*) blood pressure was affected by HFD

Rats on the high-fat diet (HFD) for 6 weeks gained more body weight (+17 %, p = 0.002, Fig. 2a; Table 2) and had an almost threefold increase (p = 0.001) of the perirenal and epidydimal fat depots compared to chow-fed control animals. The HFD had no effect on blood pressure (Fig. 2b) and heart/body weight ratio (Table 2). Under non-fasting conditions, significant differences in blood glucose, plasma insulin (p = 0.07) and triglyceride levels were not observed between groups. Only the plasma total cholesterol level was higher (p < 0.001) in HFD rats compared to control rats.

**Table 2:** Characteristics after 6 weeks of HFD versus control rats on standard chow

	Control (N = 9)	HFD (N = 9)
Body composition		
Body weight (g)	392.2 ± 6.9	459.1 ± 10.4*
Heart weight (g)	1.08 ± 0.05	1.25 ± 0.06
Heart weight/body weight (x1000)	2.67 ± 0.11	2.67 ± 0.20
Perirenal fat (g)	3.40 ± 0.31	11.88 ± 1.41*
Epidydimal fat (g)	2.83 ± 0.14	9.20 ± 1.09*
Blood/plasma characteristics		
Hematocrit (%)	51.5 ± 1.7	49.3 ± 0.8
Blood glucose (mmol/l)	6.25 ± 0.05	5.58 ± 0.80
Plasma insulin (ng/ml)	0.32 ± 0.05	0.55 ± 0.11
Total cholesterol (mmol/l)	1.69 ± 0.07	2.24 ± 0.08*
Triglycerides (mmol/l)	0.83 ± 0.13	0.81 ± 0.1

Data are expressed as mean ± SEM

* p < 0.05. HFD, high fat diet. Heart weight, perirenal fat and epidydimal fat was determined in 4 control rats and 4 HFD rats (n = 8)

**Fig. 3:**
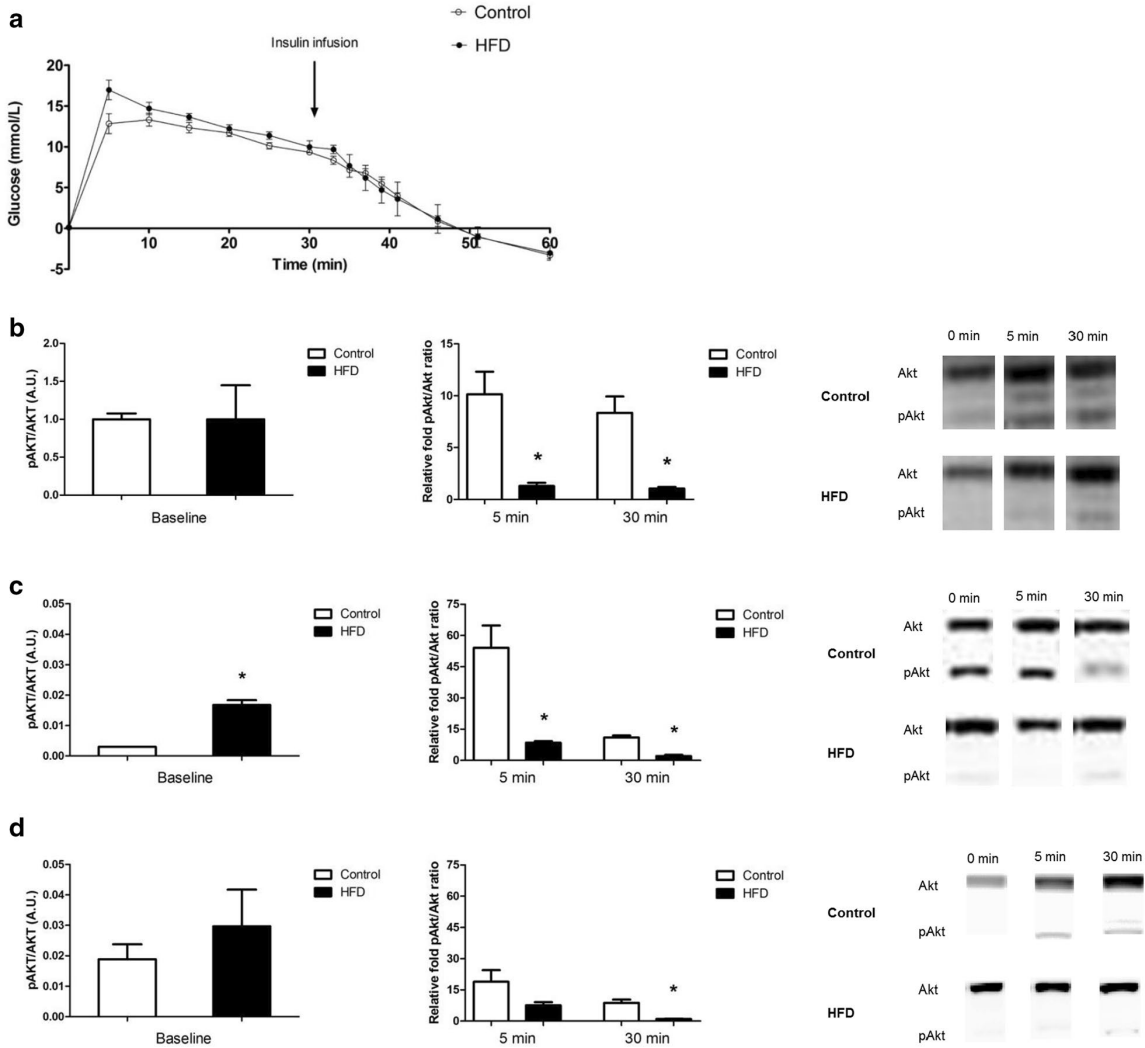
Intravenous insulin tolerance test (IVITT) in control and HFD rats. **a** Glucose level was corrected for baseline glucose level and time indicates the time after glucose infusion. No differences in glucose levels were observed between control (n = 9) and HFD rats (n = 9). Ventricular tissues (**b**), gastrocnemius muscle (**c**) and soleus muscle (**d**) were isolated at t = 0 (baseline conditions), t = 5 and t = 30 min (n = 3 per time point) after the insulin injection. The ratio of pAkt to total Akt was measured in hearts of control rats (*open bars*) and HFD rats (*filled bars*). For both cardiac and skeletal muscle tissue, Akt phosphorylation was decreased in HFD rats at 5 an 30 min after insulin infusion. For the soleus muscle, Akt phosphorylation was only significantly decreased at 30 min after insulin infusion. For all isolated tissues, representative Western Blots are shown. Data are expressed as mean ± SEM, *p < 0.05 diet effect

In fasted rats, however, insulin levels were higher in HFD rats compared to control rats (0.76 ± 0.26 and 0.16 ± 0.03 ng/ml, respectively, p < 0.05). Nonetheless, significant differences between control and HFD rats were not observed during the insulin tolerance test (Fig. 3a).The insulin-induced phosphorylation of Akt in the cardiac muscle (Fig. 3b), gastrocnemius muscle (Fig. 3c) and soleus muscle (Fig. 3d) was sharply reduced in HFD rats compared to control rats (p = 0.02, p < 0.001 and p = 0.001 respectively). The collective findings indicate that feeding rats a HFD for a relatively short period already induced marked abdominal adiposity and early stage of insulin resistance, but no hypertension.

### Cardiac function

**Fig. 4:**
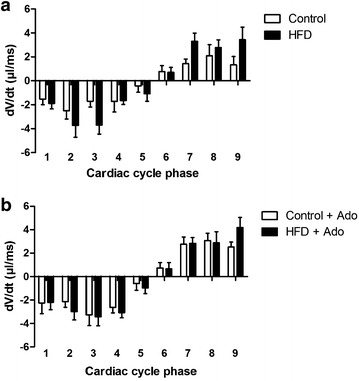
Cardiac cycle left ventricular dV/dt values for control and HFD rats. **a** First derivatives of LV volume with respect to time for control (n = 9, *open bars*) and HFD rats (n = 9, *filled bars*) are presented for nine phases of cardiac cycle during baseline conditions. **b** dV/dt values are presented for cardiac cycle during adenosine infusion. The negative dV/dt values correspond to systole and positive values correspond to diastole. Data are expressed as mean ± SEM, differences in absolute dV/dt values for the cardiac cycle phase were tested with ANOVA; *p < 0.05

Baseline cardiac function, as assessed by MRI, was comparable between HFD and control rats (Table 3). Adenosine infusion evoked a significant drop in heart rate and a rise in end-diastolic left ventricular (LV) volumes, resulting in an increased SV in both groups. Ejection fraction (EF) was comparable for both groups under baseline conditions as well as after adenosine infusion. The change of LV volume as a function of time (dV/dt) during the cardiac cycle was not significantly different between the control and HFD rats for all cardiac cycle phases (Fig. 4). However, absolute dV/dt values during systole and diastole tended to be increased for the HFD rats compared to control rats at baseline (p = 0.082), whereas no such effect was seen during adenosine infusion (p = 0.919).

**Table 3:** Left ventricular geometry and function determined by cine MRI of control rats and rats on HFD for 6 weeks

	Control		HFD	
	Baseline (N = 9)	Adenosine (N = 9)	Baseline (N = 9)	Adenosine (N = 9)
Heart rate (bpm)	341 ± 13	312 ± 26*	386 ± 7	320 ± 16*
EDV (µl)	320 ± 15	380 ± 23*	295 ± 21	353 ± 36*
ESV (µl)	104 ± 12	108 ± 22	79 ± 11	79 ± 14
SV (µl)	216 ± 14	271 ± 34*	216 ± 20	274 ± 36*
EF (%)	67 ± 3	70 ± 7	73 ± 4	76 ± 4
Wall thickness (mm)				
LV free wall	1.49 ± 0.29		1.64 ± 0.24	
Septum	1.58 ± 0.22		1.67 ± 0.21	

Data are expressed as mean ± SEM

*HFD* high fat diet, *EDV* end-diastolic volume, *ESV* end-systolic volume, *SV* stroke volume, *EF* ejection fraction

* p < 0.05 adenosine effect

In addition diastolic wall thickness of the LV free wall and the septum was not different between HFD rats and control rats (Table 3).

### Myocardial microvascular perfusion

**Fig. 5:**
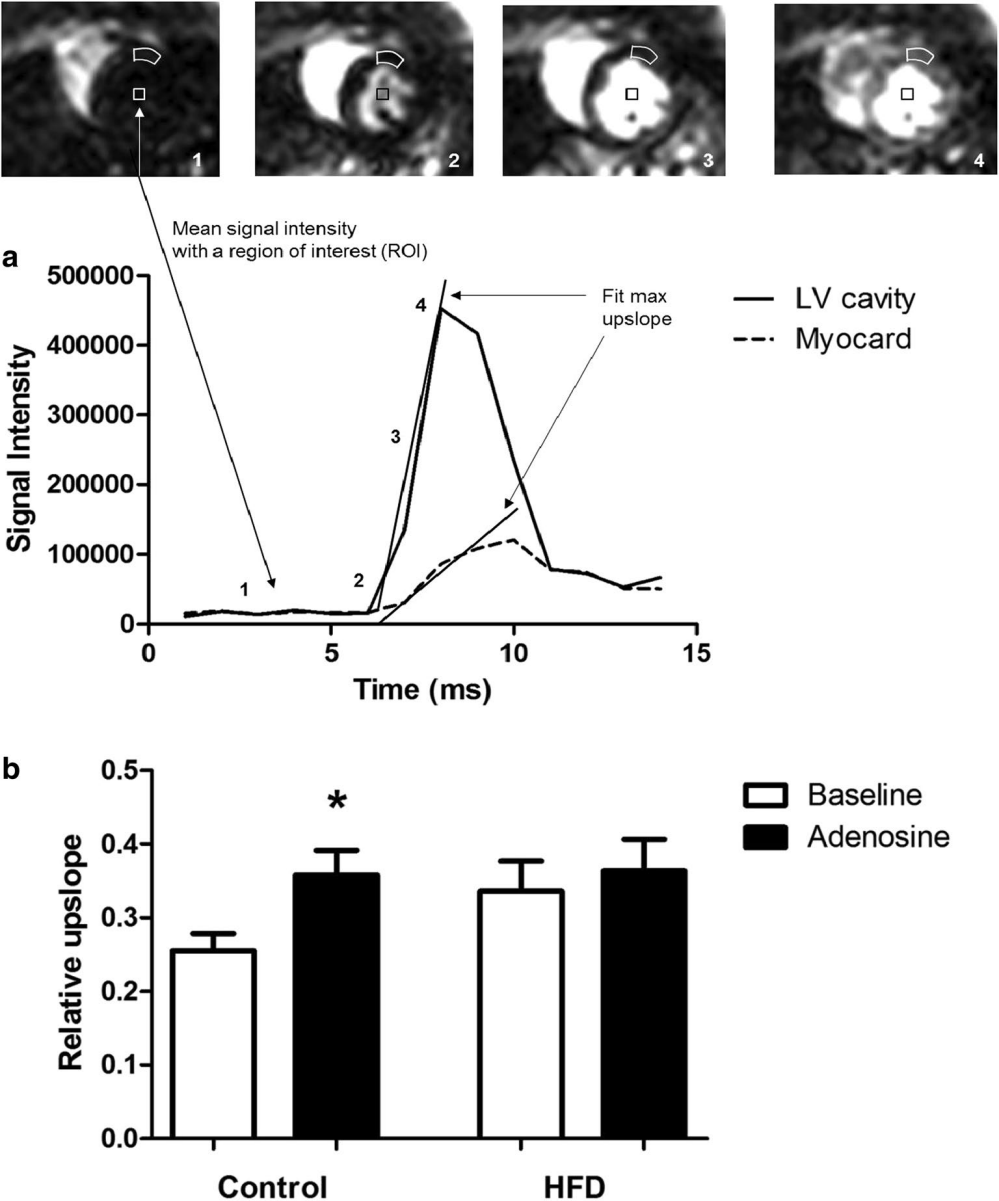
Myocardial perfusion measurements with first-pass perfusion MR imaging. **a** Signal intensity-time curve of LV cavity and myocardium derived from the mean signal intensity within regions of interest (ROI) measured in an MR image of the heart. Relative upslope was determined by dividing maximal upslope of the myocardium by maximal upslope of the LV cavity. **b** Semi-quantitative myocardial perfusion values (relative upslope) in LV myocardium of control rats (n = 9) and rats on HFD (n = 9) were presented during baseline conditions and adenosine infusion. Data are expressed as mean ± SEM, *p < 0.05 adenosine effect

The effect of HFD on coronary microvascular perfusion under baseline conditions and following adenosine infusion was assessed by contrast-enhanced first-pass MRI by calculating the relative upslope, obtained from the signal intensity-time curve of the LV cavity and the LV myocardium upon contrast Gadobutrol injection (Fig. 5a). In control rats adenosine induced a robust increase in the relative upslope of the signal intensity-time curve (+40 %, p = 0.02), reflecting an increase in coronary microvascular perfusion (Fig. 5b). The adenosine response was greatly reduced (+8 %) in rats on a HFD for 6 weeks. The blunted adenosine response in the myocardium of HFD rats appeared to be mainly due to an already increased perfusion at baseline (+32 % in HFD vs control), although this difference did not reach the level of statistical significance (p = 0.11).

### Glycocalyx properties

**Fig. 6:**
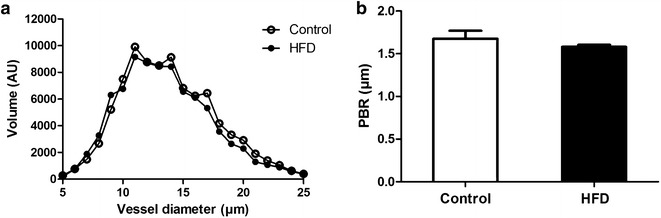
Imaging of the gastrocnemius muscle microcirculation to measure glycocalyx barrier properties. **a** Volume was determined for all vessel diameters (5–25 µm) for control rats (n = 9) and HFD rats (n = 9) and are not significantly different (p = 0.82). **b** No significant differences are found between the PBR of control rats (1.68 ± 0.09) and HFD rats (1.58 ± 0.02). Data are expressed as mean ± SEM, *p < 0.05

As the cardiac microcirculation cannot be visualised directly in vivo, the effect of HFD on microcirculatory properties was assessed by sidestream darkfield (SDF) imaging of the gastrocnemius muscle of the same animals. SDF imaging in combination with Glycocheck software allows determination of vessel density and the perfused boundary region (PBR), a functional measure of glycocalyx integrity [35]. In the gastrocnemius muscle the percentage of perfused vessels (84 and 77 % for HFD and control rats) and number of perfused vessels (1012 ± 64 and 1020 ± 77; p = 0.93), their cumulative volume (arbitrary units: 84619 ± 689 and 89525 ± 714 for HFD and control rats, p = 0.815), as well as the volume distribution for vessels ranging from 5 to 25 µm in diameter (Fig. 6a), did not differ between HFD and control rats. The PBR of rats receiving HFD amounted to 1.58 ± 0.02 µm and was comparable to rats on normal chow (1.68 ± 0.09 µm, p = 0.34, Fig. 6b). The collective findings suggest that 6 weeks of HFD did not result in rarefaction or loss of glycocalyx integrity in skeletal muscle.

**Fig. 7:**
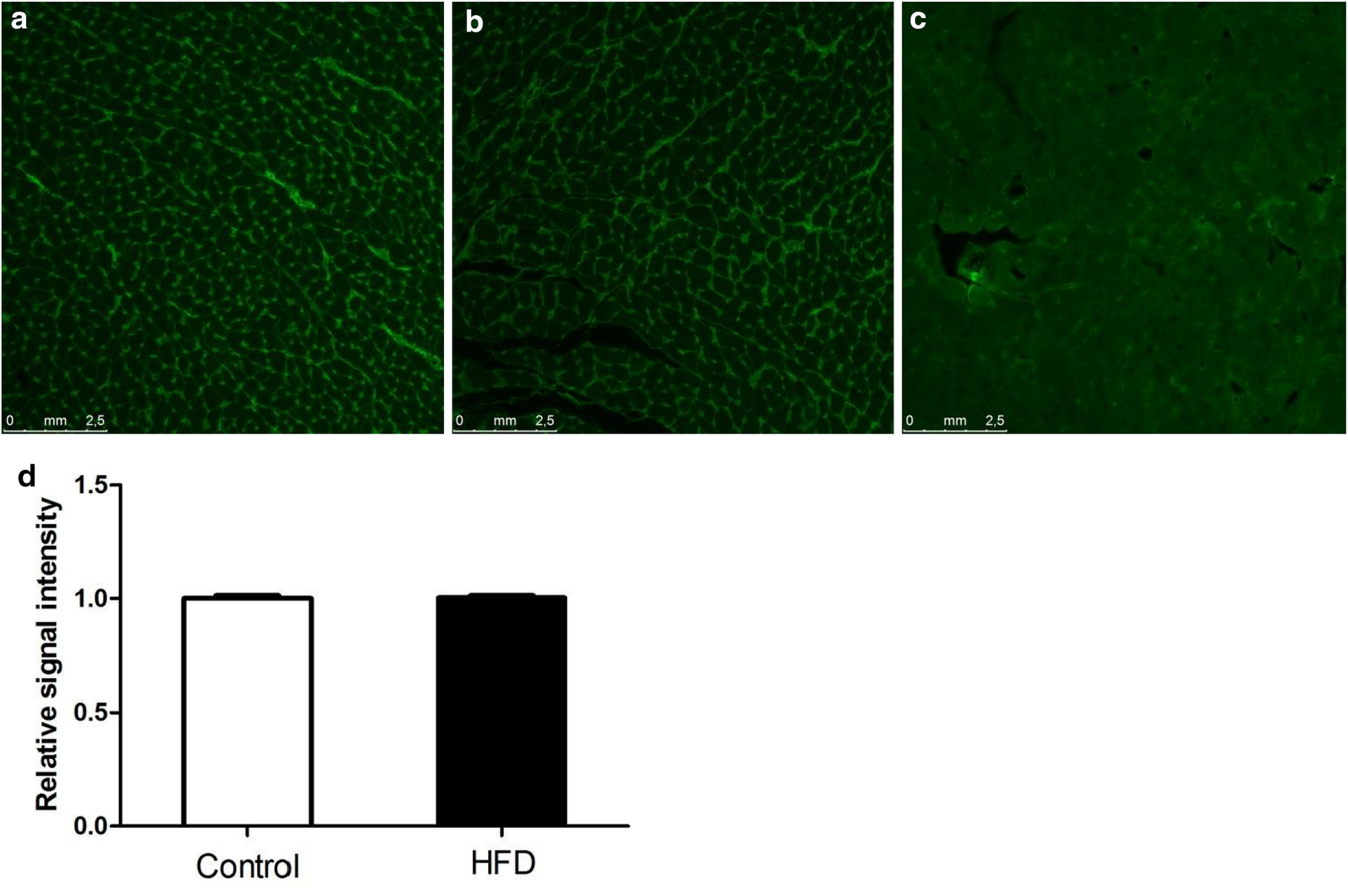
Evaluation of cardiac myocyte size and signal intensity in control and HFD rats. A wheat germ agglutinin (WGA) FITC-labeled lectin staining was performed on heart tissue of control rats and HFD rats (**a**), The intensity of WGA-staining was measured in control rats (n = 4) and in HFD rats (n = 4; **b**). **c** As a positive control, cardiac tissue from hyaluronidase-treated rats was used, a clearly visible reduction in capillary WGA-staining intensity was shown. **d** Cardiac staining intensity did not differ between control and HFD rats

Next, the intensity of WGA-staining of the capillary glycocalyx was used to determine if the glycocalyx of the cardiac microcirculation was affected by the HFD. As a proof of concept, we first showed that intravenous application of hyaluronidase, an enzyme known to degrade the glycocalyx [39], resulted in a clearly visibly reduction in capillary WGA-staining intensity (Fig. 7c). However, using automated software analysis differences in cardiac staining intensity between HFD and control rats were not detected (Fig. 7a, b, d), suggesting that the HFD did not compromise cardiac glycocalyx properties to a significant extent.

### Cardiac remodeling

To explore if HFD led to cardiac hypertrophy, tissue sections were stained with wheat germ agglutinin FITC-labeled lectin. Differences in cardiomyocyte size between control rats and HFD rats, however, were not observed. This finding is supported by the unaltered expression of cardiac hypertrophy markers like atrial natriuretic factor (ANF) and α-skeletal actin (αSKA) (Table 4). In line with the absence of changes in cluster of differentiation 68 (CD68) mRNA expression, CD68 immunostaining revealed no significant increase of macrophage infiltration in heart tissue of rats on HFD (18 ± 6 vs 14 ± 6 CD68-stained cells per mm^2^ LV tissue for HFD and control rats, p = 0.66; images not shown). The expression levels of the inflammation marker monocyte chemoattractant protein-1 (MCP1) showed no significant upregulation in HFD rats compared to control rats either. Oil-red-O staining showed no significant lipid accumulation in the myocardium of HFD rats relative to control rats (images not shown). Expression of angiopoietin-like protein 4 (Angptl4) was not affected, while pyruvate dehydrogenase kinase 4 (PDK4) tended to increase (p = 0.11). The mRNA levels of the endothelial activation genes intercellular adhesion molecule (ICAM), vascular adhesion molecule (VCAM), endothelial nitric oxide synthase (eNOS), were also unaltered in HFD rats compared to control rats (Table 4).

**Table 4:** Cardiac expression levels of genes involved in endothelial activation, inflammation, metabolism and cardiac hypertrophy in control and HFD rats after 6 weeks of high fat diet feeding

	Control (N = 8)	HFD (N = 9)	P value
Endothelial activation			
ICAM	1.0 ± 0.19	0.67 ± 0.13	0.13
VCAM	1.0 ± 0.22	0.79 ± 0.14	0.43
eNOS	1.0 ± 0.17	0.86 ± 0.14	0.52
Inflammation			
MCP1	1.0 ± 0.26	1.08 ± 0.41	0.88
CD68	1.0 ± 0.25	0.65 ± 0.07	0.17
Metabolism			
PDK4	1.0 ± 0.17	1.94 ± 0.51	0.11
Angptl4	1.0 ± 0.35	1.13 ± 0.31	0.79
Hypertrophy			
ANF	1.0 ± 0.39	0.45 ± 0.26	0.25
αSKA	1.0 ± 0.24	1.36 ± 0.42	0.49

Data are expressed relative to control rats (chow) as mean ± SEM

* p < 0.05

## Discussion

In this study, we demonstrated that feeding rats a high fat diet for 6 weeks already leads to disturbances in coronary microcirculatory perfusion as determined by contrast enhanced first-pass perfusion MRI at baseline and after an adenosine challenge. Importantly, the HFD-induced changes in cardiac microcirculatory perfusion are associated with central obesity and an impaired cardiac insulin sensitivity, but not with hypertension or signs of cardiac remodeling. The collective findings suggest that changes in coronary microcirculatory function represent one of the earliest obesity-related functional abnormalities in the cardiac muscle.

### Diet-induced obesity

Diet-induced obesity is associated with dyslipidemia, glucose intolerance and insulin resistance in rats [25, 40–42]. In the present study, 6 weeks of HFD led to a marked increase in abdominal fat depots and a rise in serum total cholesterol levels, but not in a rise of serum triglyceride, insulin or glucose levels as measured under non-fasting conditions. After overnight fasting, however, insulin levels were higher in the rats on a HFD compared to chow-fed rats. Moreover, insulin signaling in the heart and skeletal muscle of HFD rats was already disturbed, as evidenced by the reduced insulin-mediated phosphorylation of Akt in these tissues. Protein Kinase B (PKB) or Akt is an important signaling protein in the intracellular insulin pathway. In myocytes, phosphorylation of Akt (pAkt) triggers translocation of the glucose transporter GLUT4 to the sarcolemma, which enables glucose to enter the myocytes [43]. Reduced insulin-mediated Akt phosphorylation reflects impaired signaling downstream of the insulin receptor and, thus, reduced insulin sensitivity of the cardiac muscle [44, 45].

### Cardiac function and cardiac remodeling

In the present study, 6 weeks of HFD feeding was not associated with marked changes in cardiac function. At baseline, the ejection fraction was comparable between HFD and control rats, suggesting that systolic function was not compromised. Moreover, adenosine infusion led to a comparable decrease in heart rate and increase in end-diastolic LV volume in control and HFD rats. Intriguingly, at baseline the rate of LV volume change across the cardiac cycle (dV/dt) tended to be increased for HFD rats in both systole and diastole suggesting that, if anything, baseline cardiac function is enhanced, rather than reduced, after 6 weeks of HFD. In literature inconsistent findings have been reported as far as the effect of HFD on myocardial function is concerned, with some studies reporting reduced function [25, 46], unchanged function [42] and improved function [21], respectively. In this context it is of note that one study reported that functional outcome was dependent on the exact composition of the HFD diet [42].

In the current study the HFD did not affect blood pressure as measured in awake animals. Furthermore, there were no indications for HFD-induced cardiac remodeling. HFD for 6 weeks did not induce cardiac hypertrophy as assessed by heart weight to body weight ratio, LV wall thickness, cardiomyocyte size and expression of hypertrophic marker genes. Accordingly, the collective findings indicate that the observed changes in coronary microvascular perfusion in HFD rats cannot be explained by HFD-induced changes in blood pressure, cardiac function and cardiac remodeling.

### Myocardial microvascular perfusion

In the present study we used first-pass contrast-enhanced MRI to assess the effects of diet on the coronary microcirculation. In chow-fed rats adenosine resulted in a robust >40 % rise in coronary perfusion as assessed by the normalized relative upslope of the signal-time curve, reflecting an adequate hyperaemic response. In contrast, in the myocardium of rats fed a HFD for 6 weeks the hyperaemic response was completely blunted. The absence of the hyperaemic response in the myocardium of HFD rats appeared to be due largely to an already increased coronary perfusion at baseline. Others reported that coronary flow reserve (CFR) in rats was not affected at all after 4 weeks of HFD [25]. In mice, long-term HFD feeding (24 weeks) was associated with a reduction in CFR [22]. Likewise, a reduction in CFR was observed in patients with established type 2 diabetes, but not in insulin resistant patients [23]. Others reported a decline in CFR in obese, insulin resistant patients [19, 24], which was largely explained by concurrent hypertension-related increases in basal myocardial blood flow [19]. Indeed, baseline myocardial blood flow was similar in healthy controls and diabetic patients without hypertension [47]. In obesity-prone pigs feeding a HFD for 10 weeks did not affect blood pressure and basal myocardial flow. After 14 weeks, however, when insulin resistance was established, basal myocardial flow was increased, presumably in response to an increased systolic function [48].

As reported above, in the present study, the HFD was not associated with a rise in blood pressure, excluding hypertension as a cause for the increase in baseline coronary perfusion. Although at baseline heart rate was slightly higher in HFD rats than in control rats (+13 %, Table 3), the difference in coronary perfusion at baseline was much more pronounced (+32 %), implying that the difference in cardiac work cannot explain the increased coronary perfusion in HFD rats at baseline.

In theory, HFD-induced endothelial dysfunction due to endothelial activation, increased microvascular permeability, or glycocalyx damage may account for the observed perfusion defects [24, 49]. Using tracer dilution techniques, we recently demonstrated that in mice suffering from severe diabetes the endothelial glycocalyx was compromised at the systemic level [50]. In the present study, however, signs of endothelial activation or HFD-mediated inflammation were not found, as the expression of endothelial activation markers (ICAM, VCAM) remained constant and influx of inflammatory cells in the myocardium was not observed. In line with this, coronary arteriolar vasomotor activity was found to be enhanced in rats on a HFD [20, 21], suggesting that endothelial function is not compromised under these conditions. Even in rat models of established diabetes (Zucker Diabetic rats) no signs of coronary endothelial dysfunction were observed [51].

It was previously shown that hyperlipidemia induces degradation of the glycocalyx and increases vascular wall adhesiveness [52]. As damage to the cardiac endothelial glycocalyx cannot be measured directly, possible systemic effects of HFD on glycocalyx barrier properties were measured by visualising the microcirculation of the gastrocnemius muscle by means of SDF imaging, which allows estimation of the so-called perfused boundary region (PBR). The PBR is considered to reflect glycocalyx integrity and is based on the notion that loss of glycocalyx allows for deeper penetration of erythrocytes into the endothelial lining, resulting in higher PBR values [38]. In an earlier study on impairment of skeletal muscle endothelial glycocalyx barrier properties in diet-induced obesity in mice, it was shown that the PBR in the skeletal muscle microcirculation was significantly increased 6 weeks after HFD already [13]. The present findings indicate that PBR was not (yet) affected in rats after 6 weeks of HFD. The difference in glycocalyx barrier properties observed between the two studies can possibly be explained by the level of insulin resistance that is attained by the HFD in rats and mice. In contrast to the present study, Eskens et al. [13] observed a marked rise in plasma glucose and insulin levels in mice in response to HFD, indicative of established insulin resistance. In the current study, however, we observed no differences in plasma levels, although the reduced insulin-stimulated AKT phosphorylation in muscle points to an already impaired insulin sensitivity.

Theoretically, changes in PBR can be masked by microvascular rarefaction. However, the absence of changes in total vessel volume and in vessel diameter distribution argue against such a masking effect. Assuming that the glycocalyx properties of the gastrocnemius muscle are representative for the cardiac muscle, it must be concluded that damage to the glycocalyx of the coronary endothelium cannot explain the HFD-induced change in coronary microvascular perfusion. However, it remains to be determined if the gastrocnemius microcirculation is a good proxy for the cardiac microcirculation when it comes to glycocalyx function as, unlike the heart, the gastrocnemius muscle is inactive during the SDF measurements, contains both white and red muscle fibers, and produces energy by both aerobic and anaerobic metabolism [53]. Nonetheless, the absence of significant differences in capillary WGA-staining intensity in cardiac histological sections supports the contention that the glycocalyx of the coronary microcirculation was not damaged to a significant extent in rats fed a HFD for 6 weeks.

Alternatively, the increase in baseline myocardial perfusion in rats on a HFD can possibly be explained by a shift of myocardial substrate use towards an increase in fatty acid (FA) oxidation. Normally 60–70 % of the energy needs of the beating heart are covered by the oxidation of FA, whereas carbohydrates (mainly glucose) account for the remaining 30–40 % [54–56]. In diabetes, cardiac glucose utilization is minimal, and energy production is shifted almost exclusively towards the oxidation of fatty acids (FA) [54]. Since cardiac insulin sensitivity is decreased in the HFD rats, the heart will become even more dependent on the oxidation of FA [54]. As the oxidation of FA requires more oxygen than glucose (lower ATP/O ratio), cardiac efficiency (ratio of cardiac work to myocardial oxygen consumption) will decrease under these conditions [57]. This would fit with the observation that hemoglobin saturation, as assessed by Blood Oxygen Level-Dependent (BOLD) MRI, was decreased in the myocardium of obese, insulin resistant pigs [48, 58]. Thus, it is well conceivable that in diet-induced obese rats the myocardium may compensate for this diminished cardiac efficiency by increasing basal myocardial perfusion, thereby already consuming part of its coronary flow reserve capacity. However, further studies are warranted to establish a direct link between cardiac substrate metabolism and coronary flow reserve in the (pre-)diabetic heart.

## Conclusions

In conclusion, the present data demonstrate that changes in coronary microvascular perfusion as assessed by first-pass perfusion MRI represent one of the earliest cardiac adaptations that can be assessed non-invasively in diet-induced obesity. At this stage glycocalyx barrier properties in the gastrocnemius muscle were not affected as a consequence of the HFD challenge. The diet-induced changes in coronary microvascular perfusion occur at a time when cardiac insulin sensitivity gets compromised, but prior to the development of hypertension and cardiac remodeling, which are commonly associated with more advanced insulin resistance and type 2 diabetes. First-pass perfusion MRI may represent a valuable diagnostic tool for the early detection of cardiac microvascular perfusion abnormalities in obese, insulin resistant persons, and in patients with non-ischemic heart disease.

## Abbreviations

Akt: protein kinase B
ANF: atrial natriuretic factor
Angptl4: angiopoietin-like protein 4
αSKA: α-skeletin actin
CD68: cluster of differentiation 68
CRF: coronary flow reserve
4CH: 4-chamber
dV/dt: change of left ventricular volume as a function of time
EDV: end-diastolic volume
EF: ejection fraction
eNOS: endothelial nitric oxide synthase
ESV: end-systolic volume
FA: fatty acids
HFD: high fat diet
ICAM: intercellular adhesion molecule
IVITT: intravenous insulin tolerance test
LA: long axis
LV: left ventricular
MCP1: monocyte chemoattractant protein-1
MRI: magnetic resonance imaging
NO: nitric oxide
PBR: perfused boundary region
PDK4: pyruvate dehydrogenase kinase 4
RBC: red blood cell
ROI: region of interest
SA: short axis
SDF: sidestream dark-field
TE: echo time
TR: pulse repetition time
VCAM: vascular adhesion molecule
