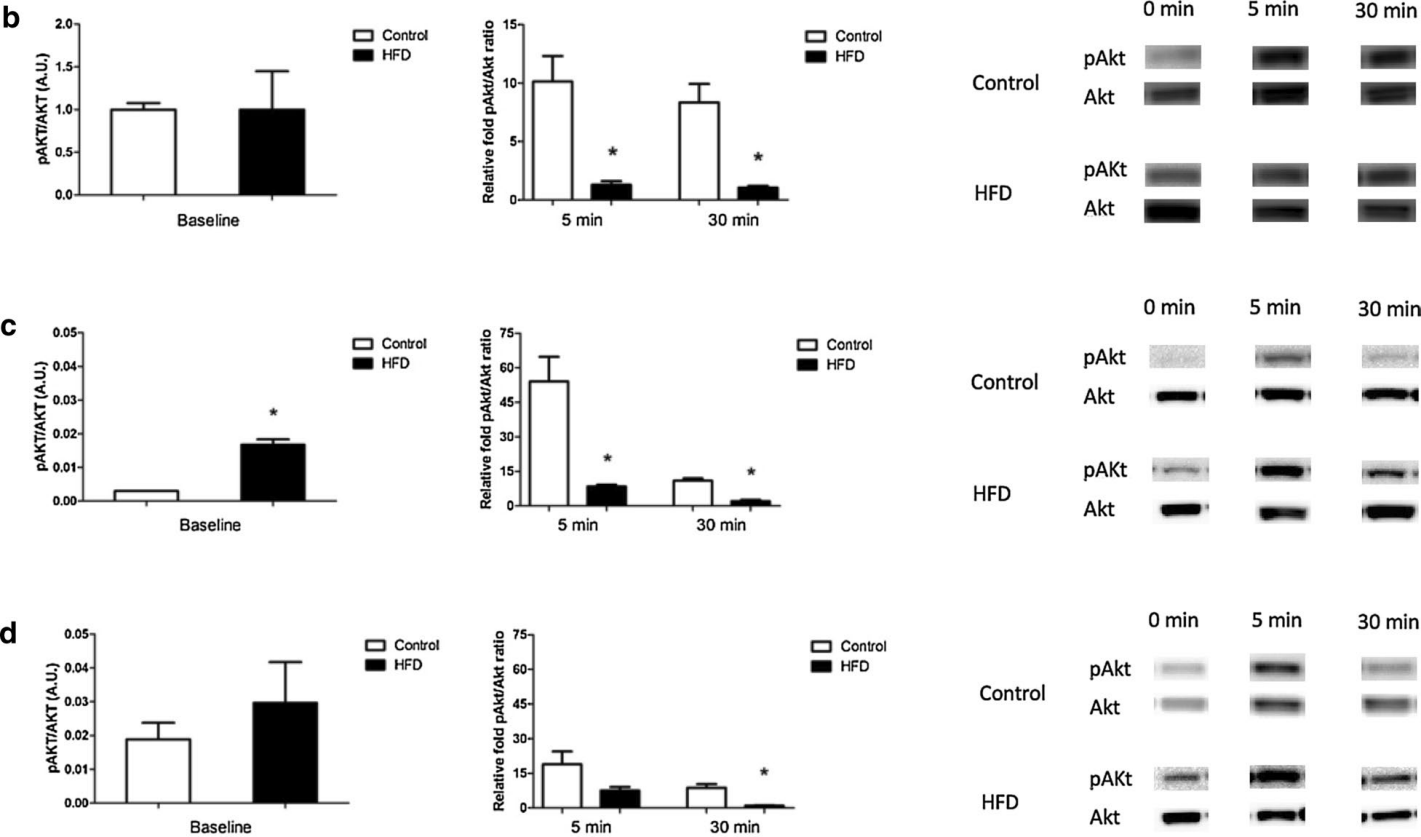# Correction to: Early impairment of coronary microvascular perfusion capacity in rats on a high fat diet

**DOI:** 10.1186/s12933-021-01239-0

**Published:** 2021-02-22

**Authors:** Judith van Haare, M. Eline Kooi, Hans Vink, Mark J. Post, Jurgen W. G. E. van Teeffelen, Jos Slenter, Chantal Munts, Hanneke Cobelens, Gustav J. Strijkers, Dennis Koehn, Marc van Bilsen

**Affiliations:** 1 Department of Physiology, CARIM School for Cardiovascular Diseases, Maastricht University, P.O. Box 616, 6200 MD Maastricht, The Netherlands; 2 Department of Radiology, CARIM School for Cardiovascular Diseases, Maastricht University, P.O. Box 616, 6200 MD Maastricht, The Netherlands; 3 Department of Cardiology, CARIM School for Cardiovascular Diseases, Maastricht University, P.O. Box 616, 6200 MD Maastricht, The Netherlands; 4 Biomedical Engineering and Physics, Academic Medical Center, P.O. Box 22700, 1100 DE Amsterdam, The Netherlands; 5 Pie Medical Imaging, P.O. Box 1132, 6201 BC Maastricht, The Netherlands

## Correction to: Cardiovasc Diabetol (2015) 14:150 https://doi.org/10.1186/s12933-015-0312-2

Following publication of the original article [1], the authors regret errors in Fig. 3b–d. In these figures, the images of the representative Akt and phospho-Akt (pAkt) signals should be replaced with the appropriate images. The representative images shown here are correct. The changes do not affect the scientific conclusion and significance of the article.